# The State Urge to be Physically Active‐Questionnaire (SUPA‐Q): Development and validation of a state measure of activity urges in patients with eating disorders

**DOI:** 10.1002/brb3.3220

**Published:** 2023-08-09

**Authors:** Lina Amin, Georg Halbeisen, Karsten Braks, Thomas J. Huber, Georgios Paslakis

**Affiliations:** ^1^ University Clinic for Psychosomatic Medicine and Psychotherapy, Medical Faculty, Campus East‐Westphalia Ruhr‐University Bochum Luebbecke Germany; ^2^ Centre for Eating Disorders Klinik am Korso Bad Oeynhausen Germany

**Keywords:** eating disorders, physical activity, psychopathology, psychotherapy, symptom assessment

## Abstract

**Objective:**

Many people, including patients with eating disorders (EDs), experience an increased urge for physical activity. “Trait”‐like activity in patients with EDs is assessed by existing questionnaires, but there are few clinically validated assessments of a “state” urge to be physically active. Here, we developed and validated the State Urge to be Physically Active‐Questionnaire (SUPA‐Q).

**Methods:**

After developing and piloting the items, *N* = 126 patients with EDs (mostly anorexia nervosa and bulimia nervosa) took part in our mixed‐longitudinal validation study with one primary assessment for all patients and a secondary assessment for a subsample of patients. Cronbach's *α* and split‐half‐methods served as measures of consistency and reliability. Correlations with other questionnaires were used to determine convergent and divergent validity, and confirmatory factor analysis was used for investigating factorial validity. We used paired‐samples *t*‐tests for repeated assessments to investigate change sensitivity.

**Results:**

We found the SUPA‐Q to be highly consistent, and reliable and to demonstrate convergent, divergent, and factorial validity. The comparison of SUPA‐Q scores from repeated assessments within a subsample of patients demonstrated the questionnaire's change sensitivity, Cohen's *d* = 0.48. Moreover, an increase in SUPA‐Q scores was associated with a less positive mood, more anxiety, more body dissatisfaction, more tenseness, less feelings of control, and more stress.

**Discussion:**

The newly developed SUPA‐Q may help to accentuate the necessity to evaluate and address the acute urge to engage in physical activity in patients with EDs in clinical practice and ultimately support tailoring treatments to patients’ unique symptom patterns. The questionnaire is available at https://doi.org/10.17605/OSF.IO/G2YBC.

## Graphical abstract

We have developed and validated the State Urge to be Physically Active‐Questionnaire (SUPA‐Q), a novel instrument to assess the acute urge (“state”) to be physically active in patients with eating disorders (EDs).

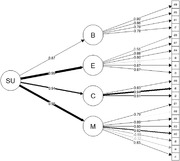

## 1 INTRODUCTION

Anorexia nervosa (AN) and bulimia nervosa (BN) are severe eating disorders (EDs) associated with the fear of gaining weight and a distorted body image (American Psychiatric Association, 2013). Patients with EDs are typically obsessively preoccupied with food and body shape or weight and engage in ritualized behaviors such as counting calories, frequent weighing, and “excessive” physical activity in both quantity (e.g., frequency) and quality (e.g., compulsiveness; Rizk et al., 2015). Physical activity broadly refers to any body movement produced by skeletal muscle contractions as part of both exercising behaviors (which are planned, structured, repetitive, and purposeful) and non‐exercising behaviors (e.g., standing, walking, or fidgeting; Caspersen et al., 1985). Up to 80% of patients across all types of EDs carry out an increased level of physical activity (Keyes et al., 2015; Shroff et al., 2006), despite experiencing fatigue and tiredness (Casper et al., 2020; Gümmer et al., 2015).

Increased physical activity levels can be observed in numerous healthy and clinical populations (Stults‐Kolehmainen et al., 2022). In patients with EDs, high levels of exercising and non‐exercising physical activity have been associated with the severity of ED‐specific psychopathology, longer hospitalizations, and worse outcomes, including shorter times to relapse and a chronic course of the disorder (Bratland‐Sanda et al., 2010; Cook et al., 2014; Gianini et al., 2016; Peñas‐Lledó et al., 2002). High levels of (non‐exercising) physical activity in patients with EDs may not only result from neuroendocrine adaptive changes to underfeeding (Casper, 2006) but also from efforts to work off calories and regulate affect. Indeed, exercising primarily initiated for weight regulation and body shaping displays the strongest association with ED‐specific psychopathology (Mond & Calogero, 2009). The association between the drive for thinness and physical activity is also particularly prevalent in patients with high levels of chronic negative affectivity (Vansteelandt et al., 2007), who may engage in exercising due to its anxiolytic effect (Greenwood et al., 2005). It has been speculated that patients with EDs thus gradually develop an urge for physical activity, with individuals initially engaging in exercising to maximize weight loss, which then transitions into a coping strategy for alleviating negative affect and body image concerns, ultimately evolving into an autonomous and compulsive motivation for any form of physical activity (Meyer et al., 2011; Peñas‐Lledó et al., 2002; Rizk et al., 2020).

Activity levels and underlying motivations in patients with EDs are usually assessed through self‐report questionnaires, interviews, or actigraphy (Belak et al., 2017; Di Lodovico & Gorwood, 2020; Gianini et al., 2016; Klein et al., 2007; O'Hara et al., 2016; Schmidt et al., 2017; Solenberger, 2001; Zipfel et al., 2013). There are questionnaires assessing the “addictive”‐ and “obsessive/compulsive”‐like components of increased physical activity in patients with EDs, for example, the Exercise Dependence Scale (Hausenblas & Downs, 2002) and the Commitment to Exercise Scale (CES; Davis et al., 1993). These scales conceptualize patterns of physical activity in patients with EDs in terms of a stable “trait,” but they do not depict momentary motivation states such as desires, wants, urges, and related constructs that may better explain fluctuations in physical activity and emotional experiences (Stults‐Kolehmainen et al., 2020). Indeed, patients with EDs, especially those with AN and BN, often report an acute “urge” to engage in physical activity (Casper et al., 2020; Graap et al., 2018). Consistent with recent theoretical models, we refer to the urge for physical activity as an intense “affectively charged motivation state” (Stults‐Kolehmainen et al., 2020) that is characterized by changes in the cognitive, emotional, and behavioral/motor levels. There are findings for a putative biological determinant of this momentary urge in terms of an inverse association to peripheral leptin levels (Ehrlich et al., 2009), although learning and other mechanisms may be equally at play (Stults‐Kolehmainen et al., 2022).

Thus far, only a few validated and established instruments assess motivation states related to physical activity. The recently developed Cravings for Rest and Volitional Energy Expenditure (CRAVE) scale was designed as a general measure of motivation states for both physical activity and sedentary behaviors (Stults‐Kolehmainen et al., 2021). Its items capture current wants and desires to engage in specific behaviors (e.g., exert muscles, sit down), and the scale shows good psychometric properties in non‐clinical populations. However, the CRAVE scale has not been validated in patients with EDs, and it does not cover several of the cognitive, emotional, and specific behavioral aspects related to activity urges that are reported by patients with EDs, in addition to the associated burden. For example, “burn some calories” is only included as an unscored filler item, and guilt for not being active is not assessed (Graap et al., 2018). Thus, there is a continuing need to develop an instrument to assess acute activity urges in patients with EDs.

The objectives of the present study were (a) to develop an appropriate instrument to assess the acute urge (“state”) to be physically active in patients with EDs and (b) to test the psychometric properties and validate the newly developed questionnaire in a clinical sample of patients with EDs. Therefore, we first piloted an initial version of the State Urge to be Physically Active‐Questionnaire (SUPA‐Q) created by an expert panel and then validated the SUPA‐Q in a clinical sample of patients with EDs (mostly AN and BN) in a mixed‐longitudinal study design. Specifically, we sought to assess the SUPA‐Q's consistency, reliability, factorial validity, and construct validity in patients with EDs. We expected acute activity urges to be mirrored by cognitive, emotional, and motor components, as well as aspects of burden. We also expected that the SUPA‐Q would be correlated with trait‐like assessments of physical activity drives (convergent validity), but not with general impulsivity (divergent validity; note that we did not include the CRAVE scale as it was unavailable at the time of conceptualizing this project). In addition, a subsample of patients was asked to complete the SUPA‐Q on separate days—characterized by different levels of potential triggers of activity urges—to evaluate the SUPA‐Q's sensitivity to change; in this regard, we expected the presence of triggering events to increase acute activity states (i.e., scores) in the SUPA‐Q.

## 2 METHOD

### 2.1 Development of the SUPA‐Q

We followed a deductive approach to develop items and validate the questionnaire (Burisch, 1984). Extending our previous work (Paslakis et al., 2017), an international panel of long‐term experts in diagnosing and treating EDs (psychologists and physicians) created and evaluated items to assess the urge to be physically active within several rounds of discussions. Ultimately, the panel agreed on the content and formulation of a set of 21 items that contained statements of the cognitive, emotional, and behavioral/motor aspects of the immediate urge to engage in physical activity and the associated subjective burden. The set included three inverted items to allow identifying (and excluding) participants showing patterns of response tendencies. The items were originally conceptualized in English and translated by the research team into German in a single‐stage process prior to piloting.

We administered the initial German version of the SUPA‐Q among healthy, adult volunteers (i.e., without an ED), which we recruited among faculty, friends, family, and students of our university. *N* = 52 (40 women, 12 men, *M*_age_ = 27.32, age range: 20 to 59 years) rated the statements of the SUPA‐Q items on a 5‐point Likert‐like scale from 1 (*not at all*, 0%) to 5 (*a great deal*, 100%). An additional single‐item (SI) rating provided at the end of the questionnaire queried participants’ overall immediate urge to engage in physical activity (from 1, *absent*, to 5, *very strong*). The instructions stated that participants should use the items to indicate their urge to engage in physical activity **right now, in this moment** (bold print used in the original instructions). Afterward, we collected basic demographic information (age, sex, language ability, level of education) and asked participants to (optionally) provide written feedback to identify drawbacks (e.g., ambiguous item formulations) in open text fields.

The initial SUPA‐Q showed high levels of internal consistency (Cronbach's α = .92) and (split‐half) reliability (*r*_12c_ = .90). However, there were eight items that participants either identified as ambiguous or that had low item‐selectivity values (i.e., corrected item‐total correlations < .30). Based on these outcomes, we linguistically and formally revised these items and created the final form of the SUPA‐Q (for full German and English versions, see https://doi.org/10.17605/OSF.IO/G2YBC).

### 2.2 Validation of the SUPA‐Q

To validate the German SUPA‐Q, we recruited a clinical sample of inpatients with EDs at an EDs specialty clinic, the “Klinik am Korso,” Bad Oeynhausen, Germany. The study used a mixed‐longitudinal design with one primary assessment for all patients and a secondary assessment for a subsample of patients. Patients were eligible for inclusion if they reported AN or BN diagnosis and were 18 years of age or older. All individuals were diagnosed according to ICD‐10 criteria (International Statistical Classification of Diseases and Related Health Problems: Tenth Revision) (World Health Organization, 2004) based on the clinical judgment of long‐term experts in the diagnosis and treatment of EDs (trained physicians and psychologists), and diagnoses were validated by means of peer review (therapists’ meetings). Data collection commenced in November 2021 and was set to last up to one year. The study (including the pilot) was reviewed and approved by the Ethics Committee of the Ruhr‐University Bochum's Medical Faculty at Campus East‐Westphalia (AZ 2021–799, July 19, 2021). Informed consent was obtained from all patients. The validation has been preregistered at https://aspredicted.org/YSZ_P53.

We planned one primary assessment for all patients and a secondary assessment for a subsample of patients to evaluate the SUPA‐Q's reliability, validity, and, change sensitivity. Newly admitted patients were approached for assessment #1 on Saturdays, after lunch, during the first 2 weeks of treatment in the clinic and were asked to complete the SUPA‐Q as well as additional questionnaires (see Measures section). During the first 2 weeks, patients are required to not leave the clinic grounds, engage in exercise, or skip meals. Moreover, patients are weighed daily (each morning, including on weekends) and are thus repeatedly exposed to an event commonly seen as stressful in the context of EDs. Weighing is mandatory, although patients may choose that weighing results are not disclosed to them. We hypothesized that repeated weight assessments would increase the urge for physical activity, and therefore specifically chose the first 2 weeks of treatment for our initial assessment. Consequently, assessment #2 for evaluating change sensitivity was scheduled to take place at least 1 week after assessment #1, at which point daily weighing usually ceased. Patients who agreed to participate in the assessment #2 were asked to complete the SUPA‐Q a second time. The data of this subsample were used to evaluate the SUPA‐Q's change sensitivity.

### 2.3 Measures

At each assessment, patients completed the German SUPA‐Q and provided socio‐demographic data (age, sex [female, male, diverse], language ability, level of education) in a clinical report form. Patient weight and diagnoses were validated through the comparison with clinic records. Diagnoses were made by long‐term experts in the field of EDs. Patients further provided their (self‐reported) weight, height, time since last meal, time since last physical activity, current medication, number of previous treatments, additional disorders, number of current days in treatment, number of experienced weighing days, and time (days) since last weighing. Moreover, patients indicated their current emotional state concerning (positive) mood, anxiety, body dissatisfaction, tenseness, sense of control, and level of stress, using six independent visual analogue scales ranging from 0 (*not at all*) to 100 (*very strong*).

On assessment #1, patients completed three additional questionnaires to examine the SUPA‐Q's construct (i.e., convergent and divergent) validity. Specifically, patients filled out the German CES (Zeeck et al., 2017), which assesses obsessive‐compulsive aspects of physical activity, and the German Exercise Dependence Scale (EDS; Müller et al., 2013), which assesses the dependent‐like phenotype of physical activity; both questionnaires were used for assessing convergent validity and were expected to correlate with the SUPA‐Q, even though they evaluate physical activity as a “trait” rather than a “state” (as is the case with our newly developed SUPA‐Q). The German short form of the Barratt Impulsiveness Scale (BIS‐15; Meule et al., 2011), which measures trait impulsivity, was expected to not correlate with the SUPA‐Q and was thus used to test for divergent validity.

For exploratory reasons, all patients also completed the German Eating Disorder Examination‐Questionnaire (EDE‐Q; Hilbert et al., 2007) at assessment #1, in order to explore the associations of the severity of the ED with the SUPA‐Q.

### 2.4 Statistical analyses

We used χ^2^ tests of independence and univariate analysis of variance (ANOVA) to compare categorically and continually measured patient characteristics and questionnaire responses, respectively. Means, SDs, difficulties (scale mean divided by scale range), and selectivity (corrected item‐total correlations) were used to evaluate the SUPA‐Q items individually. Cronbach's *α* and split‐half correlation (with Spearman–Brown correction) were used to evaluate scale consistency and reliability. Because we assumed that activity urges cause subjective burden as well as cognitive, emotional, and specific behavioral experiences, and that the items capturing these related dimensions should factor accordingly, a confirmatory factor analysis (CFA) with maximum likelihood estimation (rather than an exploratory approach) was used to evaluate the SUPA‐Q's factorial validity (Levine, 2005). Thus, we specified a hierarchical model with the state urge (SU) as second level factor and cognitive aspects (C), emotional aspects (E), motor aspects (M), and burden (B) of state activity urges as first‐level predictors of item variance. We evaluated the fit using the comparative fit index (CFI), the root mean square error of approximation (RMSEA), and the standardized root mean square residual (SRMR). According to convention, values > 0.90, < 0.10, and < 0.08 indicate acceptable levels of fit (Hu & Bentler, 1999). We also compared the hierarchical model to a simple, single‐factor solution (Kliem et al., 2016). Moreover, construct validity was examined using bivariate correlations of questionnaire mean scores (with 95% confidence intervals, CI), and sensitivity to change was evaluated with paired‐samples *t*‐tests.

The significance level for all analyses was set at *p* ≤ .05. Post hoc pairwise comparisons report Bonferroni‐adjusted *p*‐values for multiple comparisons. Effect sizes are reported as *η^2^* and Cohen's *d*. All analyses were performed using SPSS Statistics version 28 for Windows (IBM Corp., 2021), except for the CFAs that were calculated in R 4.2.2 (R Core Team, 2022) using package lavaan 0.6‐12 (Rosseel, 2012). We used case‐wise exclusion in analysis with missing data. All analyses were conducted after terminating data collection.

## 3 RESULTS

### 3.1 Patient demographics

After 1 year of data collection (November 2021 through October 2022), *N* = 126 patients (117 women, 9 men, *M*_age_ = 26.3, age range: 18 – 65 years) participated in the study, with *n* = 64 diagnosed (according to the ICD‐10) with AN, *n* = 38 with BN, *n* = 11 with unspecified EDs, and *n* = 1 with binge eating (without fulfilling the diagnostic criteria for BN or binge‐ED). Twelve patients self‐reported their ED diagnosis (five AN, four BN, and three with AN or BN subtypes), but we could not confirm their diagnoses as we had no access to their patient records. Two of these patients also were not able to provide information on their weight. However, as we had no doubt about the presence of an ED in these patients (the clinic only accepts and treats patients with EDs), along with the fact that the exclusion of these 12 patients from statistical analyses did not change the pattern of results, all patients were included in the analyses in the end. One participant did not answer three of the EDE‐Qs rating questions, but the mean scores were nevertheless computable. The data were otherwise complete.

Table 1 summarizes the group demographics. Sex, language skills, and level of education were similarly distributed across ED groups, all *p*s > .40. Univariate ANOVAs revealed the expected group differences in body mass index (BMI), *F*(3, 120) = 22.78, *p* < .001, *η^2^* = .36, with a lower BMI among patients with AN, compared to patients with BN and unspecified EDs, *p*s < .001; other *p*s > .19. There was also a main effect for days in treatment, *F*(3, 122) = 3.01, *p* = .031, *η^2^* = .07, but pairwise comparisons between groups were not significant, all *p*s > .14. Age, number of weighing days, number of days since last weighing, hours since last meal, hours since last physical activity, and the number of previous treatments did not differ between groups, all *F*s < 2.5, *p*s > .06.

**TABLE 1:** Patient demographics (mean ± *SD*) stratified by diagnosis groups.

Variable	AN	BN	UED	Other	Total
n (f/m/d)	69 (63/6/0)	42 (40/2/0)	11 (10/1/0)	4 (4/0/0)	126 (117/9/0)
Language^a^					
Native	67	39	11	4	121
Fluent	2	2	0	0	4
Basic knowledge	0	1	0	0	1
Education					
< 12 years	17	14	4	0	35
≥ 12 years	52	28	7	4	91
Age (years)	25.59 ± 9.52	26.64 ± 8.01	29.27 ± 14.40	25.75 ± 8.88	26.27 ± 9.48
BMI (kg/m^2^)	16.37 ± 2.34	22.07 ± 4.92	21.61 ± 4.96	21.11 ± 6.21	18.83 ± 4.61
Days treatment	6.75 ± 8.02	11.43 ± 13.19	12.09 ± 14.19	20.50 ± 31.69	9.21 ± 11.92
Weighing days	5.10 ± 3.13	6.52 ± 5.64	6.00 ± 2.32	7.25 ± 5.32	5.72 ± 4.17
Days since last weighed	0.49 ± 1.22	1.19 ± 1.90	1.45 ± 1.86	0.75 ± 1.50	0.82 ± 1.57
Hours last meal	0.31 ± 0.55	0.28 ± 0.27	0.25 ± 0.25	0.13 ± 0.10	0.29 ± 0.44
Hours last activity	9.54 ± 17.50	5.50 ± 8.43	4.77 ± 5.93	4.25 ± 5.19	7.61 ± 14.07
#previous treatm.	1.60 ± 2.25	1.50 ± 4.40	2.00 ± 2.41	2.25 ± 0.96	1.62 ± 3.11

Abbreviations: AN, anorexia nervosa; BN, bulimia nervosa; ED, UED, unspecified eating disorder; f/m/d, female, male, diverse; BMI, body mass index.

^a^ German language ability (native vs. fluent vs. basic knowledge of German) was defined according to self‐evaluation.

### 3.2 Item characteristics

Table 2 displays means, standard deviation, item difficulties (scale mean divided by scale range), and selectivity (corrected item‐total correlations) for the 21 SUPA‐Q items and the SI overall rating. Difficulties ranged from 0.29 to 0.70, and except for three items, absolute item‐total correlation consistently scored above the conventional threshold of 0.30.

**TABLE 2:** Item means, standard deviations, difficulty, and selectivity.

Item	Label	M	SD	Difficulty	Selectivity
i1	Urge to move	3.63	1.28	0.66	0.80
i2	Burn calories	3.31	1.41	0.58	0.83
i3	Guilt	3.25	1.47	0.56	0.84
i4*	Keeping still	3.35	1.33	0.59	−0.28
i5	Feel good	3.21	1.35	0.55	0.84
i6	Compensate	3.40	1.44	0.60	0.88
i7	No strength	2.53	1.48	0.38	0.63
i8*	Ignore	2.76	1.12	0.44	−0.53
i9	Happy	2.17	1.12	0.29	0.17
i10	Body concern	3.09	1.49	0.52	0.77
i11	Burdened	3.04	1.46	0.51	0.76
i12	Must move	3.10	1.41	0.53	0.87
i13	Feeling forced	2.51	1.36	0.38	0.76
i14	Calming	3.02	1.36	0.51	0.75
i15	Ignoring harm	2.45	1.47	0.36	0.73
i16	Preoccupation	3.22	1.51	0.56	0.85
i17	Worrying	2.98	1.51	0.50	0.85
i18*	Sitting/standing still	3.23	1.44	0.56	−0.03
i19	Against will	2.62	1.38	0.41	0.67
i20	Relief	3.78	1.42	0.70	0.54
i21	Importance	2.34	1.31	0.34	0.73
SI	Overall urge	3.42	1.19	0.60	0.86

*Note*: Negative correlations are expected for inverted items (marked with an asterisk); these should be recoded before aggregating the scale.

### 3.3 Internal consistency and reliability

After recoding the inverted items 4, 8, and 18, the 21 SUPA‐Q items achieved, according to convention, high internal consistency of Cronbach's *α* = .95 in the total sample. The split‐half reliability (with Spearman–Brown correction) was similarly high at *r*_12c_ = .95. Both values improved, compared to the pilot investigation.

### 3.4 Factorial validity

**FIGURE 1:**
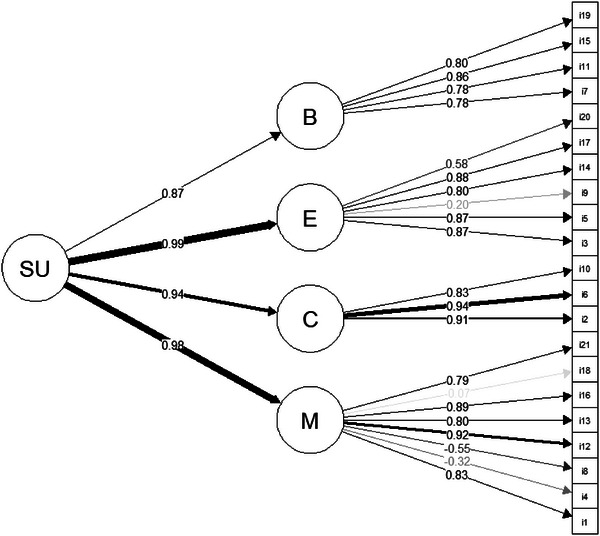
Factor model with standardized loadings for the State Urge to be Physically Active‐Questionnaire items. SU = state urge to be physical active, B = burden, E = emotional aspects, C = cognitive aspects, M = motor aspects. Bolder and darker lines represent stronger loadings.

The CFA revealed acceptable fit parameters for the hypothesized second‐order general factor model (state urge, SU; see Figure 1), with first‐order factors for the cognitive aspects (C), emotional aspects (E), motor aspects (M), and burden (B) of state activity urges (CFI = 0.91, RMSEA = 0.09, SRMR = 0.06). A single latent‐factor model, in which all items loaded onto the SU factor, provided worse fit indices (CFI = 0.87, RMSEA = 0.11, SRMR = 0.07) and was therefore rejected.

### 3.5 Construct validity

CES, EDS, and BIS‐15 questionnaires were aggregated according to their specifications (Cronbach's α = .93, .95, and .77, respectively). We then calculated the correlations of the SUPA‐Q mean score with the other test scores to evaluate convergent and divergent validity. As expected, the SUPA‐Q mean was positively correlated with CES and EDS scores, *r* = .80, 95% CI [0.72; 0.85] and *r* = .76, 95% CI [0.67; 0.82], respectively. Moreover, the SUPA‐Q and BIS‐15 scores were not significantly correlated, *r* = −.02, 95% CI [−0.16; 0.14].

We also evaluated the validity of the single (global) item regarding the immediate urge to be active, which showed similar levels of convergent and discriminant validity. The SI correlated positively with the CES and EDS scores, *r* = .72, 95% CI [0.63; 0.80] and, respectively, *r* = .69, 95% CI [0.58; 0.77], and did not correlate with the BIS‐15 score, *r* = −.01, 95% CI [−0.18; 0.16].

### 3.6 Sensitivity to change

Forty patients (37 women, three men, *M*_age_ = 24.8, age range: 18–48) completed the second SUPA‐Q assessment at least 1 week after providing their initial ratings, with *n* = 35 providing their second ratings on a non‐weighing day (average days since last weighing: *M* = 2.80, *SD* = 1.91). As hypothesized, a paired‐samples *t*‐test confirmed higher activity urge ratings during the initial assessment, compared to the second assessment (*M*_#1_ = 2.99, *SD* = 1.06 vs. *M*_#2_ = 2.72, *SD* = 1.01), *t*(39) = 3.04, *p* = .004, *d* = 0.48, 95% CI [0.15; 0.81]. The effect remained significant after excluding patients who provided their second ratings on a weighing day, *t*(34) = 2.55, *p* = .015, *d* = 0.43, 95% CI [0.08; 0.77].

### 3.7 Additional analyses

Because previous research suggested that activity urges are positively associated with ED severity, we additionally evaluated the associations between SUPA‐Q mean scores and different clinical parameters. First, we examined correlations between SUPA‐Q mean scores and patient BMI, both across all patients and within diagnostic groups (i.e., patients with validated AN and BN diagnoses). However, the correlations were not significant, *r*s = −.06 (all patients), 0.01 (AN only), and 0.01 (BN only), all *p*s > .52.

Second, we examined associations between the SUPA‐Q and the EDE‐Q as a psychometric measure of ED severity. The EDE‐Q items group into the four subscales Restraint Eating (Cronbach's *α* = .90), Eating Concern (0.76), Weight Concern (0.80), and Shape Concern (0.89). A multiple correlation of the four subscales with the SUPA‐Q was significant, *adj. R* ^2^ = .52, *F*(4, 121) = 35.46, *p* < .001. However, standardized partial correlation coefficients of the subscales showed that only Restraint was positively associated with the SUPA‐Q, *β* = .46, *p* < .001, but neither Eating Concern, *β* = .11, *p* = .21, Shape Concern, *β* = .09, *p* = .49, nor Weight Concern, *β* = .17, *p* = .24.

Third, we compared SUPA‐Q scores between groups of patients with validated AN (*n* = 24 restrictive and *n* = 25 active subtype) and BN (*n* = 37) diagnoses. Descriptively, patients with active AN showed higher state activity urges than patients with restrictive AN and patients with BN (*M_ANa_* = 3.42, *SD* = 0.77 vs. *M_ANr_* = 3.08, *SD* = 0.91 vs. *M_BN_* = 2.93, *SD* = 0.94). However, likely due to the low power of the comparisons (*1−β* = .50 for a medium‐sized effect), the overall group differences were not significant, *F*(2, 83) = 2.25, *p* = .11.

Last, we examined correlations between patients’ current affective states, the SUPA‐Q, and other questionnaires (see Table 3). An increase in the acute urge to be active was associated with a less positive mood, more anxiety, more body dissatisfaction, more tenseness, less feelings of control, and more stress. Notably, and although trait activity urges were also correlated with affective states, correlations with the SUPA‐Q mean scores were consistently higher, emphasizing the momentary nature of the questionnaire. SUPA‐Q SI scores showed consistent correlational patterns, although their magnitudes were likely reduced due to the lower reliability of the SI measure.

**TABLE 3:** Correlations of affective states with the SUPA‐Q mean, single item (SI), and trait measures related to physical activity (CES, EDS).

State items	SUPA‐Q mean	SUPA‐Q SI	CES	EDS
Mood	−0.46**	−0.38**	−0.38**	−0.33**
Anxiety	0.46**	0.37**	0.35**	0.32**
Body dissatisfaction	0.49**	0.37**	0.39**	0.33**
Tenseness	0.50**	0.40**	0.41**	0.35**
Sense of control	−0.22*	−0.14	−0.10	−0.03
Level of stress	0.41**	0.24**	0.27**	0.23*

*Note*: CES, Commitment to Exercise Scale; EDS, Exercise Dependance Scale; SUPA‐Q, State Urge to be Physically Active‐Questionnaire.

* Correlation is significant at the 0.05 level (two‐tailed).

** Correlation is significant at the 0.01 level (two‐tailed).

## 4 DISCUSSION

While a number of self‐report questionnaires have been developed, implemented, validated, and published to serve in the assessment of “excessive” physical activity (“trait”) in patients with EDs, there have been only a few validated and published instruments to assess the acute urge to be physically active (in terms of a modifiable “state”; Stults‐Kolehmainen et al., 2021). The present project's goal was to systematically develop, implement, and test the psychometric properties of a self‐report “state” questionnaire including items referring to the cognitive, emotional, and motor aspects of the acute urge to engage in physical activity in patients with EDs.

We developed the SUPA‐Q, with 21 items and an additional SI global rating, based on expert consensus to capture a state characterized by cognitive, emotional, and motor aspects as well as the associated psychological burden. After piloting and reformulating the items, the SUPA‐Q was validated in a clinical sample of patients with mostly AN and BN. The scale proved to be highly consistent, reliable, and demonstrated convergent, divergent, and factorial validity. Most importantly, a comparison of separate days with different triggers of activity urges (e.g., mandatory daily weighing) among a subsample of patients displayed the hypothesized change in SUPA‐Q scores, demonstrating the newly developed scale's change sensitivity. Taken together, these findings point to good psychometric properties of the SUPA‐Q.

In clinics specialized in the treatment of EDs, patients may find themselves in a life‐threatening state, for example, due to massive underweight and/or other ED‐associated conditions (shifts in electrolytes, alterations in blood count, etc.). Before hospitalization, some patients get involved in excessive physical activity (e.g., running multiple kilometers each day or doing extensive sets of sit‐ups). In the initial phases of standard treatment, the goal is regular participation in supervised meals to stabilize weight and continuously increase weight during treatment. To ensure weight stabilization and continuous increase, patients are not allowed to engage in physical activities in the first weeks of treatment, for example, patients are not meant to leave the ward and are required to abstain from calorie‐consuming activities, like sporting exercises. These restrictions are tough, especially for those patients who had prior engaged in extensive physical trainings. Patients then often complain about a persistent inner “urge” to be in constant motion (Graap et al., 2018). Terms like sport or exercise “addiction” are often mentioned in the context of physical activity serving as affect regulation in patients with EDs. In some patients, physical activity may indeed phenotypically resemble an “addiction” (craving the activity, loss of control over the behavior, habituation/tolerance and the need for constant intensification of the activity, symptoms of “withdrawal” when the activity is reduced or ceased, a great deal of time being spent with physical activity accompanied by the neglect of other aspects of life, and persistent activity despite clear evidence of harmful consequences). At the same time, there are also phenomenological characteristics of “compulsion,” distinguishable in the physical activity behavior of patients: perseverative thoughts with regard to activity that may in fact be experienced as distressing or even irrational, leading to anxiety when trying to refrain from such thoughts and behaviors associated with physical activity.

Physical activity urges and other motivation states relevant to explaining fluctuations in physical activity and respective emotional experiences are present in various populations (Stults‐Kolehmainen et al., 2020). While systematic syntheses of the relevant literature are ongoing (Stults‐Kolehmainen et al., 2022), we must note that, at least in patients with EDs, disorder‐specific treatments rarely explicitly address this common phenomenon. No specific therapeutic options are available to address a raised urge to be active, although the condition is perceived as being very unpleasant. There are some published programs of supervised physical activity for patients with EDs (Achamrah et al., 2016; Dittmer et al., 2018), but, especially in patients with AN, even supervised and well‐planned physical activity is not feasible during the first several weeks of inpatient treatment due to significant underweight and the need to build‐up rather than consume calories. There is also no approved medication; the antipsychotic olanzapine is thought to alleviate some of the obsessive‐compulsive cognitions in AN and is therefore suggested as an option in some patients suffering from an unbearable urge (Hillebrand et al., 2005). However, symptom relief is not always the case clinically (Graap et al., 2018). Intended for the rather later stages of treatment, after having reached a certain weight gain and stabilization, controlled exercise programs for patients with AN are well feasible. Supervised physical activity according to a plan is already considered and offered as part of a multimodal treatment of AN (Schlegel et al., 2015). To alleviate the acute urge to be physically active, we previously implemented a novel virtual reality (VR)‐based intervention (Paslakis et al., 2017). In that study, we have shown that our paradigm of VR jogging was able to reduce the acute urge to engage in physical activity in women with EDs. Future research may determine if similar approaches are applicable to other populations experiencing increased activity urges (e.g., exercise addiction, akathisia, or restless legs syndrome).

Finally, we note some limitations. Although the SUPA‐Q displayed good psychometric properties overall, the CFA showed that some SUPA‐Q items present with low factor loadings, suggesting that further refinement of formulations for some items or their removal could be justified. For the present validation, we nevertheless included these items in all analyses as their removal would require re‐assessing the scale's validity in another sample. Future studies will need to explore the validity of a reduced SUPA‐Q scale, including the stability of the observed factor structure in further samples.

We also still need to assess the prognostic validity of the SUPA‐Q for treatment outcomes and/or establish cut‐off values for alarming levels of activity urges. This will be aided by validating the questionnaire in further clinical and non‐clinical samples. Additionally, activity urges vary by time of day in non‐clinical populations (Budnick et al., 2023), which reinforces the need to explore and compare the magnitude and fluctuation of SUPA‐Q scores in future investigations with clinical and non‐clinical populations. Our scale requires further validation in patients with EDs against other recently established measures not included in the present study (i.e., the CRAVE scale; Stults‐Kolehmainen et al., 2021). While CRAVE items targeting sedentary behaviors (*rest* subscale) are not explicitly covered in the SUPA‐Q, the *move* subscale shows considerable overlap with some SUPA‐Q items and should correlate positively. Last, it is important to note that the SUPA‐Q's sensitivity to change has thus far only been examined by comparing measurements taken several days apart. However, state‐like properties suggest that activity urges, and thus SUPA‐Q scores, may change in an even shorter time frame and in response to current events, which future studies will still need to explore.

## 5 CONCLUSION

This study presents a new measure of state‐like acute activity urges showing initial validity in patients with EDs. The SUPA‐Q may help to accentuate the necessity to evaluate and address the urge to engage in physical activity in patients with EDs in clinical practice. Assessing activity urges could help identify treatment targets and tailoring treatments to patients’ unique symptom patterns.

## AUTHOR CONTRIBUTIONS

**Lina Amin**: Conceptualization; formal analysis; investigation; writing—original draft. **Georg Halbeisen**: Conceptualization; data curation; formal analysis; methodology; visualization; writing—original draft; writing—review and editing. **Karsten Braks**: Resources; writing—review and editing. **Thomas J. Huber**: Resources; writing—review and editing. **Georgios Paslakis**: Conceptualization; methodology; supervision; writing—original draft; writing—review and editing.

## PUBLIC SIGNIFICANCE STATEMENT

Increased physical activity urges can be observed in numerous healthy and clinical populations. The strong urge for physical activity has been linked to more severe symptoms in persons with EDs. Here, we developed a novel questionnaire for assessing acute activity urges in patients with EDs. Findings from our study show that the questionnaire works as intended. The new questionnaire may help researchers and clinicians to identify and address acute activity urges during treatment for disordered eating.

## CONFLICT OF INTEREST STATEMENT

The authors have no competing interests to report.

### PEER REVIEW

The peer review history for this article is available at https://publons.com/publon/10.1002/brb3.3220

## Data Availability

The data that support the findings of this study are available from the corresponding author upon reasonable request.
